# Calcium Acetate or Calcium Carbonate for Hyperphosphatemia of Hemodialysis Patients: A Meta-Analysis

**DOI:** 10.1371/journal.pone.0121376

**Published:** 2015-03-23

**Authors:** Yong Wang, Guoqiang Xie, Yuanhang Huang, Han Zhang, Bo Yang, Zhiguo Mao

**Affiliations:** 1 Division of Hemodialysis, CPLA 422 Hospital, Guangdong, China; 2 Kidney Institute of CPLA, Division of Nephrology, Changzheng Hospital, Second Military Medical University, Shanghai, China; 3 Division of Nephrology, General Hospital of Guangzhou Military Region, Guangdong, China; 4 Department of Hepatic Surgery, Eastern Hepatobiliary Surgery Hospital, Second Military Medical University, Shanghai, China; Sao Paulo State University, BRAZIL

## Abstract

**Background:**

High levels of serum phosphorus both at baseline and during follow-up are associated with increased mortality in dialysis patients, and administration of phosphate binders was independently associated with improved survival among hemodialysis population. Calcium-based phosphate binders are the most commonly used phosphate binders in developing countries for their relatively low costs.

**Objectives:**

To compare the efficacy and safety between calcium carbonate and calcium acetate in the treatment of hyperphosphatemia in hemodialysis patients.

**Methods:**

PubMed, EMBASE, Cochrane Library, Google scholar and Chinese databases (Wanfang, Weipu, National Knowledge Infrastructure of China) were searched for relevant studies published before March 2014. Reference lists of nephrology textbooks and review articles were checked. A meta-analysis of randomized controlled trials (RCTs) and quasi-RCTs that assessed the effects and adverse events of calcium acetate and calcium carbonate in adult patients with MHD was performed using Review Manager 5.0.

**Results:**

A total of ten studies (625 participants) were included in this meta-analysis. There was insufficient data in all-cause mortality and cardiovascular events for meta-analysis. Compared with calcium carbonate group, the serum phosphorus was significantly lower in calcium acetate group after4 weeks’ administration (MD -0.15 mmol/L, 95% CI -0.28 to -0.01) and after 8 weeks’ administration (MD -0.25 mmol/L, 95% CI -0.40 to -0.11). There was no difference in serum calcium levels or the incidence of hypercalcemia between two groups at 4 weeks and 8 weeks. No statistical difference was found in parathyroid hormone (PTH) levels or serum calcium by phosphorus (Ca x P) product. There was significantly higher risk of intolerance with calcium acetate treatment (RR 3.46, 95% CI 1.48 to 8.26).

**Conclusions:**

For hyperphosphatemia treatment, calcium acetate showed better efficacy and with a higher incidence of intolerance compared with calcium carbonate. There are insufficient data to establish the comparative superiority of the two calcium-based phosphate binders on all-cause mortality and cardiovascular end-points in hemodialysis patients.

## Introduction

Serum phosphorus in chronic kidney disease (CKD) patients accumulates with the decrease of glomerular filtration rate (GFR), which cause secondary elevation of parathyroid hormone (PTH) level and consequently reduces calcitriol concentration by inhibiting its secretion and promoting its metabolism. A growing body of evidence proved the correlation between elevated serum phosphorus, PTH and calcium in CKD patients, and increased morbidity, mortality, hospitalization rate, cost of care, as well as reduced quality of life.[1]

Phosphate binders, including non-calcium-based phosphate binders and calcium-based phosphate binder, are widely used to lower serum phosphorus levels in CKD patients and prospective cohort study proved that treatment with phosphorus binders was independently associated with improved survival among incident hemodialysis patients.[2] A meta-analysis[3] and an observational cohort study[4] recently shown non-calcium-based phosphate binders slight superior to calcium-based phosphate binders on mortality reduction in CKD patients. However, in developing countries, non-calcium-based phosphate binders (sevelamer and lanthanum carbonate) are not easily available for their high prices, and calcium carbonate and calcium acetate remain to be the most commonly used phosphate binders.[5,6] Several clinical trials and reviews compared calcium acetate and calcium carbonate in efficacy and safety issues, but no clear recommendation has been published between these two options. The aim of this meta-analysis was to assess the efficacy and adverse events of calcium-based phosphate binders (calcium acetate and calcium carbonate) in hemodialysis patients. The following items were investigated in details:

1. Mortality, cardiovascular events and fracture in patients treated with calcium acetate and calcium carbonate;
2. The serum phosphorus lowering effect of calcium acetate and calcium carbonate;
3. The impact of phosphate binders on biochemical parameters such as PTH, serum calcium, phosphorus, and Ca x P product;
4. The incidence and characteristics of the adverse events and the withdraw related to treatment of calcium acetate and calcium carbonate.

## Methods

This study was registered at PROSPERO (http://www.crd.york.ac.uk/PROSPERO_REBRANDING/display_record.asp?ID=CRD42014009636). CRD42014009636. The authors search the databases published in English (PubMed, EMBASE, Cochrane Library, Google scholar) and in Chinese (Wanfang, Weipu, National Knowledge Infrastructure of China) before March 2014. “***Calcium acetate*, *calcium carbonate and dialysis/hemodialysis***” was the basic search keywords, and the related studies and the references of literatures were checked to expand the search.

Including and excluding criteria for considering studies for this review:

Inclusion criteria

1. Types of studies: randomized controlled trials (RCTs) and the first phase of randomized cross-over studies was included. Studies published in English and Chinese were included.
2. Patients: CKD Patients on hemodialysis and older than 18 years.
3. Intervention: Compared calcium acetate and calcium carbonate for controlling hyperphosphatemia in hemodialysis patients.

Exclusion criteria

1. The participants of studies were at predialysis status or patients with kidney transplant or patients with peritoneal dialysis;
2. Retrospective, as well as nonparallel studies, were excluded.

### Study selection

Two reviewers (Y. Wang and G. Xie) independently assessed the eligibility of each article to be included in this meta-analysis, and this work was checked by another author (Z. Mao).

### Data collection process and data items

Data were extracted from each identified trial by two researchers (G. Xie and H. Zhang) with a predesigned review form (Microsoft Office Excel 2007) independently, and any disagreement was resolved by discussion. Authors of the original studies were consulted through emails for suggestions if any questions regarding the original study occurred.

The following data were extracted:

1. Basic information of studies: The authors of study, the year of publication, the design, duration and the sample size of the trials, the age and gender of the patients, dialysis model, withdraw rate;
2. The interventions: The dose and usage calcium acetate and calcium carbonate, dialysis model and time, dialysate calcium concentration;
3. Treatment effect: All-cause mortality, cardiovascular mortality, cardiovascular events and incidence of fracture; Serum phosphorus (mmol/L), serum calcium (mmol/L), Ca x P product (mmol²/L²), PTH (pg/mL);
4. Side effects: Incidence and nature of treatment-related adverse effects including gastrointestinal (gastritis, diarrhea, constipation and abdominal bloating), hypercalcemia and dropout related to treatment intolerance.

### Risk of bias

The quality of included studies were evaluated by two authors (Y. Bo and G. Xie) independently based on the standard criteria (randomization, allocation concealment, blinding, and loss to follow-up) using the modified Jadad (highest 7 points) scoring system.[7] The following were quality scoring items: (1) Was the random series generated properly? (2 = Properly with detailed description of randomization, 1 = randomized but detail not reported, 0 = inappropriate); (2) Was the allocation concealment used? (2 = Properly used, 1 = used but without details, 0 = not used) (3) Was the blinding method used? (2 = Double-blind, 1 = single-blind, 0 = open-label); (4) Were dropout and follow-up reported? (1 = Numbers and reasons reported, 0 = not reported). The publication bias was assessed by examining the funnel plot. A sensitivity analysis was performed by omitting studies one by one and investigating the influence on the overall meta-analysis estimate.

### Data analysis and statistical methods

Statistical analyses were performed with Review Manager 5.0.20 (Cochrane Collaboration, Oxford, UK). We assessed the heterogeneity of the trial results by calculating a chi-square test of heterogeneity and the *I* ^*2*^ measure of inconsistency. Dichotomous data were summarized as risk ratio (RR) and 95% confidence intervals (CIs), continuous ones as weighted mean difference (WMD) and 95% CIs as well. Random effects model was used in all analysis.

## Results

### Study characteristics

**Fig 1:**
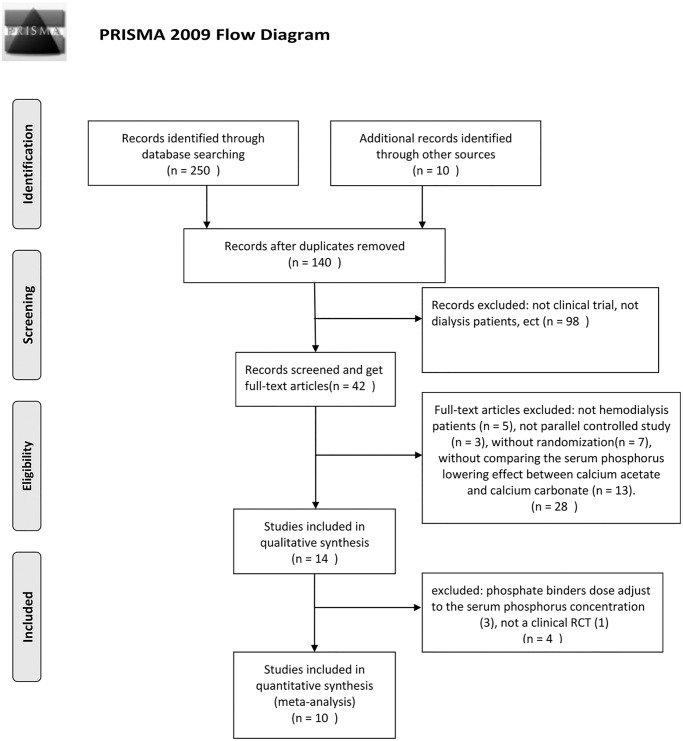
The flowchart of including and excluding literatures. (Flow Diagram).

The flow chart of including and excluding literatures was shown in Fig. 1 (Flow Diagram). After screening the abstract and excuding the studies clearly not meet the inclusion criteria, 42 full-text were obtained. Of these, 32 articles were excluded for the following reasons: 1) Participants were not hemodialysis patients (n = 5); 2) Study design were not parallel controlled study (n = 3) or without randomization(n = 7); 3) Without comparing the serum phosphorus lowering effect between calcium acetate and calcium carbonate (n = 13); 4) There were 4 studies excluded: the dose of phosphate binders were adjust to the serum phosphorus concentration (n = 3)[8–10],and not a clinical RCT (n = 1)[11]. A total of ten studies[12–21] (625 participants) were included from 260 articles retrieved. The follow up period was 4 weeks to 48 weeks and the most common phosphate binder administration period was 4 or 8 weeks. The participants were on hemodialysis for 2 to 3 times per week with the dialysate calcium concentration range from 1.25 to 1.75 mmol/L. There were 4 studies[18–21] designed as crossover trials and only 2 studies[18,20] designed as double blind. The other 6 studies are randomized open parallel controlled trials. The modified Jadad score systerm shown 4 studies[17–20] with high quality in study design(> = 4 points). The characteristics and quantities of the studies were shown in Table 1.

**Table 1:** Characteristics of the included trials.

*Trials*	*Number*	*Length*	*Mean age(year)*	*Gender male/female*	*Dialysis model*	*Dialysate calcium concentration*	*Elemental calcium dose(mg)*	*Quality grade*
Caravaca 1992	80	16 Weeks	48.5	45/35	MHD, (3 times)/week	1.62mmol/L	1500/1500	4
d’Almeida Filho 2000	52	12 Weeks	46	26/26	MHD, (3 times)/week	1.75mmol/L	1400/2500	5
Liu Q 2013	60	8 weeks	50.9	38/22	MHD, 12h/week	1.50mmol/L	942/900	2
Lu RH 2013	171	8 weeks	53.8/52.1	75/48	MHD, about 12h/week	1.25–1.50mmol/L	1000(1500)/1000(1500)^***a***^	3
Pflanz 1994	31	12 months	59.5	21/31	MHD, 12h/week	1.25mmol/L	1500/1500	4
Ring 1993	21	8 Weeks	19–75	9/6	MHD, 8–12h/week	1.74mmol/L	1440(540–2700)	5
Saif 2007	64	12 Weeks	42.6	24/17	MHD, (2–3 times)/week	NC	1014/2250	2
Tao YM 2012	60	8 Weeks	47.6	34/26	MHD, 12h/week	1.25mmol/L	1000/1050	2
Wang CF 2012	48	24 Weeks	38	30/18	MHD, 8–12h/week	1.25mmol/L	1000/1000	2
Zhang M 2013	38	4 weeks	41	22/16	MHD, (3 times) 13.5h/week	1.25mmol/L	1064/1200	3

Abbreviations: MHD, Maintenance hemodialysis; Elemental calcium dose, Elemental calcium content of phosphate binders; NC, not clear.

*^a^*. The elemental calcium dose was 1000mg and 1500mg for patients with the serum phosphorus level 1.96–2.26mmol/L, 2.27–2.75mmol/L respectively.

### Effects of interventions

#### Clinical outcomes

**Fig 2:**
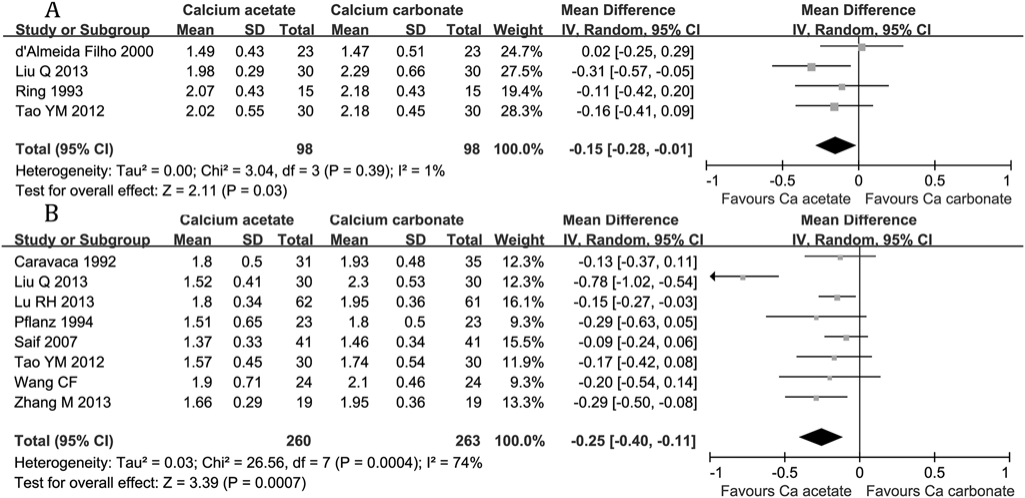
Serum phosphorus levels after 4 weeks (A) or 8 weeks (B) administration of calcium-based phosphate binders.

A total of 5 patients died after study’s randomization. Planz et al[19] reported 3 cases death all occurred on calcium acetate treatment period (crossover study): One had long-standing malignant disease, not associated with hypercalcaemia, one died of postoperative complications following a thoracotomy and the third suffered a myocardial infarction. Lu Rh et al[13] reported 2 cases of death: One in calcium acetate group suffered from sudden death and the other in calcium carbonate group died for lung infection. There was no data on fracture and cardiovascular events except the death caused by myocardial infarction.**Serum phosphorus**Compared with calcium carbonate group, there was a significant lower serum phosphorus value in calcium acetate group after 4 weeks’ treatment (4 studies, 196 participants: MD -0.15 mmol/L, 95% CI -0.28 to -0.01, Fig. 2A) and 8 weeks (8 studies, 523 participants: MD -0.25 mmol/L, 95% CI -0.40 to -0.11, Fig. 2B) respectively.

#### Serum calcium

**Fig 3:**
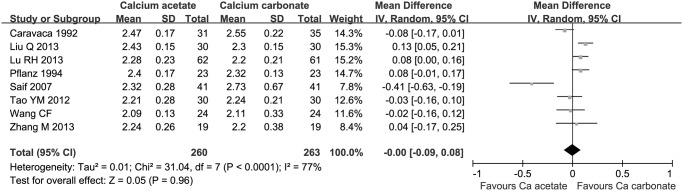
Serum calcium levels after 8 weeks administration of calcium-based phosphate binders.

There was no difference in serum calcium concentration between two groups after 4 weeks’ administration (5 studies, 319 participants, MD 0.03 mmol/L, 95% CI -0.01 to 0.07) and after 8 weeks (8 studies, 523 participants; MD 0.00 mmol/L, 95% CI -0.09 to 0.08, Fig. 3).

#### Ca x P product and PTH

**Fig 4:**
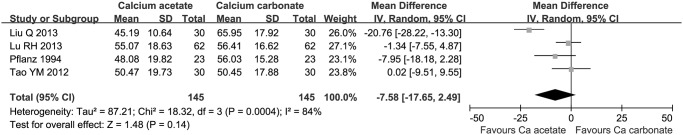
Serum calcium by phosphorus (Ca x P) products after 8 weeks administration of calcium-based phosphate binders.

**Fig 5:**
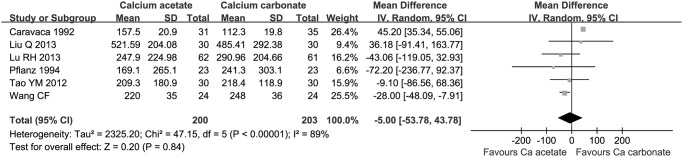
Serum PTH levels after 8 weeks administration of calcium-based phosphate binders.

There was no statistical difference on serum calcium by phosphorus (Ca x P) product (4 studies, 290 participants: MD -7.58 mmol^2^/L^2^, 95% CI -17.65 to 2.49, Fig. 4) and parathyroid hormone (PTH) levels (6 studies, 403 participants: MD -5.00 pg/mL, 95% CI -53.78 to 43.78, Fig. 5) between calcium acetate group and calcium carbonate group after 8 weeks’ administration.

### Side effects

#### Dropout related to treatment intolerance

**Fig 6:**
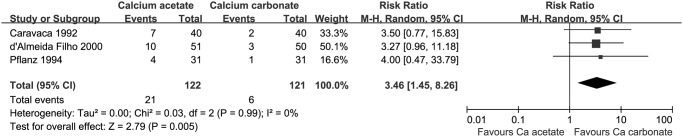
The incidences of drug intolerance in calcium-based phosphate binder groups.

There was a significantly higher incidence of intolerance with calcium acetate treatment (3 studies, 243 participants: RR 3.46, 95% CI 1.48 to 8.26, Fig. 6). Twenty-one of 122 patients (17.2%) in calcium acetate group and 6 of 121 patients (5.0%) in calcium carbonate group dropped out related to drug intolerance respectively.

#### Treatment-related adverse effects

The most common side effects reported were gastrointestinal symptoms, including epigastrium pain, bloating, nausea and anorexia. There was a trend of higher incidence of adverse gastrointestinal events in calcium acetate treated patients (11.9%) compared with calcium carbonate treated patients (5.7%), but the difference was not statistical significant (4 studies, 351 participants: RR 1.96, 95% CI 0.91 to 4.19). All side effects including gastrointestinal events were mild and easy to be treated.

#### Hypercalcemia

**Fig 7:**
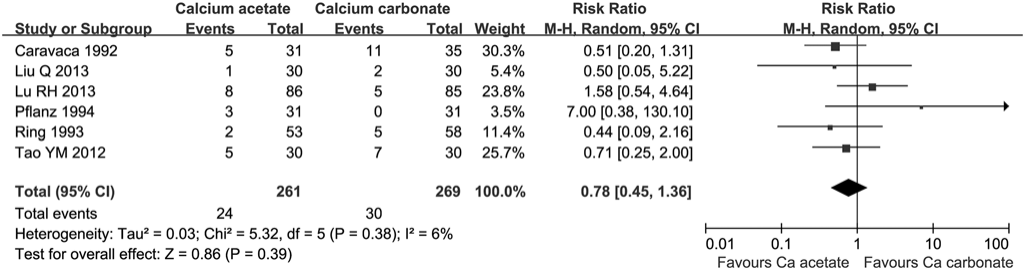
The incidence of hypercalcaemia in calcium-based phosphate binder groups.

There were 6 studies, involved 530 patients, reported the incidence of hypercalcemia. There was no significant difference for the incidence of hypercalcemia (RR 0.77, 95% CI 0.46 to 1.29, Fig. 7) between calcium acetate group (24/261, 9.2%) and calcium carbonate group (30/269, 11.2%).

### Heterogeneity and sensitivity analysis

**Fig 8:**
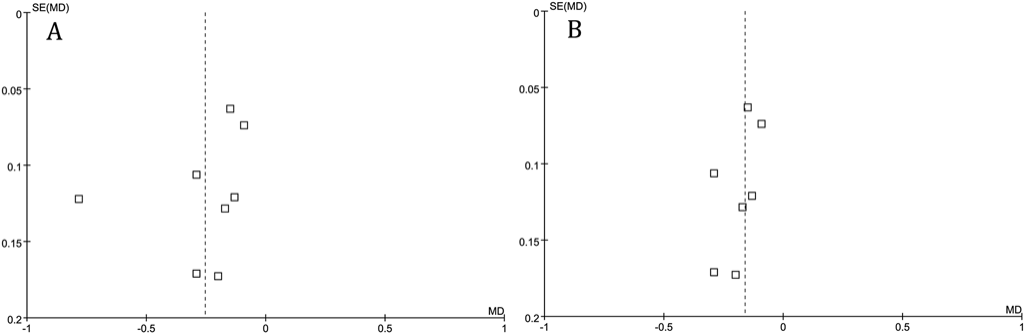
Significant visual asymmetry was revealed by the funnel plots (A) and the visual asymmetry disappeared after excluding one study with unreasonable results (B).

The funnel plots (Fig. 8A) showed significant visual asymmetry. The heterogeneity was observed in some analysis. Covariates including measures of study quality (blinding and intention-to-treat analysis), patient characteristics (baseline PTH, calcium and phosphorus concentration), and study characteristics (medication dose and study duration) were significant effect modifiers on some outcomes. We explored the potential sources of heterogeneity using subgroup analysis. However, data were insufficient to allow definitive conclusions to be drawn. For the comparison of serum phosphorus after 8 weeks’ administration, we found that the serum phosphorus did not change (from 2.28 to 2.30 mmol/L) after treatment in calcium carbonate group in one study[16], which was difficult to explain. So the authors tried to exclude this study in the serum phosphorus comparison, and the heterogeneity disappeared without changing the superior effect of calcium acetate. After exclusion of that trial mentioned above, the funnel plots (Fig. 8B) did not show significant visual asymmetry. Heterogeneity was observed in PTH value comparison (*I* ^*2*^ = 89%), the authors found that the value of serum PTH was obviously lower in Caravaca’s study.[17] So the authors tried to exclude this study in that analysis, the heterogeneity disappeared. Omitting studies one by one showed that the overall meta-analysis estimate would not be turned over by excluding any study.

## Discussion

### Summary of results and possible explanations

Serum calcium, inorganic phosphate and calcium phosphate product levels are associated with worse outcomes in patients with CKD.[1,22] Ca, P and calcium by phosphate product are useful additional variables when determining risk in chronic heart failure dialysis patients.[23] A prospective cohort study of 10,044 incident hemodialysis patients shown that treatment with phosphorus binder was independently associated with decreased mortality compared with no treatment in the intention-to-treat, as-treated, and matched analyses.[2]

This meta-analysis compared the effects and side effects of two most commonly used calcium-based phosphate binders, calcium acetate and calcium carbonate, in hemodialysis patients. To our best knowledge, this in-depth comparison involved the largest participant sample size. All-cause mortality and cardiovascular events are the final and the most important indexes for the effect and side effect of phosphate binders, however, the data were insufficient to make conclusions in our analysis as well as in other reviews.[24] This was might due to the short-term follow-up, which is difficult to show the difference on mortality and cardiovascular events. On the other hand, our review shown that 8 weeks’ administration of phosphate binders was enough to control hyperphosphatemia. (Fig. 2B)

Our review revealed that calcium acetate was more effective in reducing serum phosphorus level compared with calcium carbonate, and no difference was seen in serum calcium and PTH concentration. Although the element calcium was equal or higher in calcium carbonate group, the serum phosphorus was 0.15 mmol/L lower in calcium acetate group after an 4-week administration and 0.25 mmol/ lower after 8 weeks. This results shown the stronger effect of calcium acetate in lowing serum phosphorus, which was consistent with the previous trial[25] comparing the doses and durations of calcium acetate and calcium carbonate in achieving the target of control serum phosphorus in hemodialysis patients and consistent with the theoretical and in vitro study[26] investigating the effect of phosphate binders in inhibiting dietary phosphorus absorption in normal subjects. In a previous meta-analysis, comparison between these two calcium containing phosphate binders showed no statistical difference in reducing phosphorus level, and a small participants number involved (only 143) might attenuate the power to reveal the difference.[24]

The main side effects of both agents were gastrointestinal symptoms, including epigastrium pain, bloating, nausea and anorexia. There was a trend of higher incidence of adverse gastrointestinal events in calcium acetate group and that might lead to the higher incidence of treatment intolerance of calcium acetate. Those most reported symptoms related to the two agents disappeared after treatment withdrawal. Another announced side effect was agents relating to hypercalcaemia, our review showed hypercalcaemia occurring in 9.2% of patients in calcium acetate group and 11.2% in calcium carbonate group, without significant difference.

### Limitations of this review

Limitations of the present study including a short-term study duration (4 weeks to 12 months), the language of studies were limited to English and Chinese and studies in other languages might be missed, a small number of studies with blinding (only two trials)[18,20], relatively small number of participants (625 in total) and insufficient data on all-cause mortality and cardiovascular events. The heterogeneity and asymmetry of funnel plots was observed in the analysis, possible reasons might include the differences of study design and baseline information of participants.

## Conclusions

Based on meta-analysis of RCTs and first phase of randomized crossover studies, calcium acetate showed better effect on hyperphosphatemia control than calcium carbonate, but with a relative higher incidence of intolerance. There are insufficient data to establish the comparative superiority of the two calcium-containing phosphate binders for all-cause mortality and cardiovascular end-points in hemodialysis patients.

## Supporting Information

S1 PRISMA Checklist (DOC)
